# DIVERSITY IN ACTION: PRACTICE-BASED LEARNING FOR IMPROVING COMMUNITY HEALTH

**DOI:** 10.1093/geroni/igz038.1315

**Published:** 2019-11-08

**Authors:** Amy Thierry

**Affiliations:** Xavier University of Louisiana, New Orleans, Louisiana, United States

## Abstract

Non-traditional learning methods in diverse classrooms, particularly at HBCUs, can prepare students for successful careers in aging. In a graduate-level public health course on the social determinants of health, students engaged in an experiential learning opportunity to address health disparities within a historic Black neighborhood in New Orleans. Through a combination of classroom lectures, community integration activities, and skills-building training with faculty and public health professionals, students applied a health equity life course framework in developing, implementing, and evaluating a multi-faceted community needs assessment used to guide health promotion programming for neighborhood residents. In this example, student mastery of complex concepts was achieved through hands-on experiences that had a direct impact on the community. Moreover, a community-engaged educational environment allowed students to take ownership of their learning, develop their professional identity, as well as initiate a sustainable partnership with the community through which improvements in health can be made.

